# Diagnostic Utility of Synovial Cell Count Prior to Revision Compared to Re-Revision Arthroplasty

**DOI:** 10.3390/antibiotics15020143

**Published:** 2026-02-01

**Authors:** Jennifer Straub, Paul M. Schwarz, Laurenz Willmann, Joachim Ortmayr, Kevin Staats, Irene K. Sigmund, Reinhard Windhager, Christoph Böhler

**Affiliations:** 1 Department of Orthopaedics and Trauma Surgery, Division of Orthopaedics, Medical University of Vienna, Währinger Gürtel 18-20, 1090 Vienna, Austria; jennifer.straub@meduniwien.ac.at (J.S.);; 2 Department of Orthopaedics and Traumatology, Kepler University Hospital GmbH, Krankenhausstraße 9, 4020 Linz, Austria

**Keywords:** bone and joint infections, implant-related infections, diagnostics, PJI management, diagnostic biomarkers

## Abstract

**Background/Objectives**: The aim of this study is to investigate how the joint, the number and the type of prior revision surgeries influence the diagnostic thresholds for synovial cell count for patients who undergo their first total hip or knee arthroplasty revision compared to re-revisions, as different cutoffs might substantially influence treatment courses. **Methods**: In this retrospective single-center register analysis, data from 214 revised THAs (total hip arthroplasties) and TKAs (total knee arthroplasties) were collected, of which 103 (48.1%) have so far undergone at least one revision surgery. Diagnosis was based on the EBJIS criteria, and we identified 163 (76.2%) septic and 51 (23.8%) aseptic cases. Data on synovial cell count were collected and analyzed for their diagnostic accuracy and optimal cutoffs. For re-revisions, a covariate-adjusted ROC (receiver operating characteristic) for the joint, type of previous surgery and number of surgeries was created. **Results**: We found no significant differences in cell counts between patients before first revision compared to those undergoing re-revision for septic (*p* = 0.40) and aseptic indications (*p* = 0.84). The overall diagnostic accuracy was high for all re-revision cases, with a sensitivity of 0.86, specificity of 0.91, AUC (area under the curve) of 0.92, at an optimal cutoff value of 2439.50 G/L. As for re-revised hip joints, the optimal cutoffs were higher compared to knee joints (2439.5 G/L vs. 2626.5 G/L, hip AUC = 0.90, knee AUC = 0.93, *p* = 0.14). Furthermore, the AUCs for cell count differed significantly depending on the type of previous surgery in re-revision (*p* = 0.03). The covariate-adjusted analysis showed no significant differences compared to the unadjusted analysis. **Conclusions**: Cell count remains reliable for diagnosing periprosthetic joint infection in patients with prior revisions, with minor threshold variations from the EBJIS (European Bone and Joint Infection Society) criteria. While the type of preceding revision affects accuracy, the diagnostic value remains consistently high overall.

## 1. Introduction

Periprosthetic joint infection (PJI) is one of the leading causes of revision surgery after total hip and knee arthroplasty (THA and TKA). About 1–2% of patients after primary procedures and up to 10% after revisions are affected [1,2]. With the continued rise in primary implantations, coupled with demographic shifts, the number of PJIs is expected to increase in the near future [3,4,5]. This underlines the persistent need for the optimization of diagnostic criteria to guide management and, consequently, enhance outcomes [6].

The accurate and timely diagnosis of PJI remains challenging after primary THA and TKA, and as of today no single gold standard diagnostic test exists [7]. Furthermore, although the constant rise in primary implantations is accompanied by a corresponding increase in revision procedures [8], few studies have so far assessed the differences in diagnostic thresholds of serum and synovial markers between patients after primary implantation and those who underwent at least one previous revision. In this context, plasma fibrinogen has more proven diagnostic value than the d-dimer in diagnosis prior to re-revision surgery, but there is currently a scarcity of data when it comes to synovial biomarkers [9,10].

As the measurement of the leukocyte count in synovial fluid represents an integral part of all diagnostic workup for PJI [11,12,13], numerous studies have so far assessed optimal thresholds and have found optimal cutoffs ranging from 1100 G/L [14] to as high as 4250 G/L, with a trend towards higher values in THA compared to TKA [15]. Furthermore, a dependence on time from last surgery has been demonstrated, with cell count values normalizing not until 90 days after primary surgery [16]. Consequently, considerable variations need to be taken into account when evaluating for infection, and while the role of cell count is well studied following primary hip and knee arthroplasty [17,18], there remains a complete gap in understanding their applicability for diagnosing PJI after one or more revision surgeries.

Therefore, the aim of this study is to explore how optimal synovial cell count cutoffs vary between patients undergoing their first revision as opposed to patients who already underwent one or more TKA or THA revisions while using the EBJIS (European Bone and Joint Infection Society) guidelines as a diagnostic reference. Furthermore, differences between locations (hip or knee) are assessed, as well as the number and type of preceding revision surgery (septic or aseptic), for the diagnostic accuracy of synovial white blood cell counts in diagnosing PJI.

## 2. Results

### 2.1. Demographics and Patient Characteristics

For 111 (51.9%, 43 THAs, 68 TKAs) patients, it was the first revision procedure, whereas 103 (48.1%, 32 revised total hip arthroplasties (rTHAs), 71 revised total knee arthroplasties (rTKAs)) were previously revised at least once, with a median of 3 previous revisions (inter-quartile range (IQR) = [1; 5]). Among the 103 cases with at least one prior revision, the penultimate revision was performed for septic reasons in 62 cases (60.2%; 13 rTHAs, 49 rTKAs) and aseptic ones in 41 cases (39.8%; 19 rTHAs, 22 rTKAs). We found no significant differences between the two groups regarding demographics (cf. Table 1). Among the 163 patients fulfilling the EBJIS criteria, histological findings were given as proof of infection in 130 cases and positive cultures in 33 cases, with some patients meeting more than one confirmatory criterion. The culture results for both groups are given in Table 2.

### 2.2. First Revision, Re-Revision, and the Influence of the Number of Previous Revisions

We found no significant difference in the cell count values between patients after primary arthroplasty and those with at least one prior revision surgery, for both septic (*p* = 0.40, 71,221.89 ± 74,565.83 G/L vs. 58,643.75 ± 110,236.70 G/L) and aseptic cases (*p* = 0.84, 2036.72 ± 3008.75 G/L vs. 1889.59 ± 2331.1 G/L).

**Figure 1:**
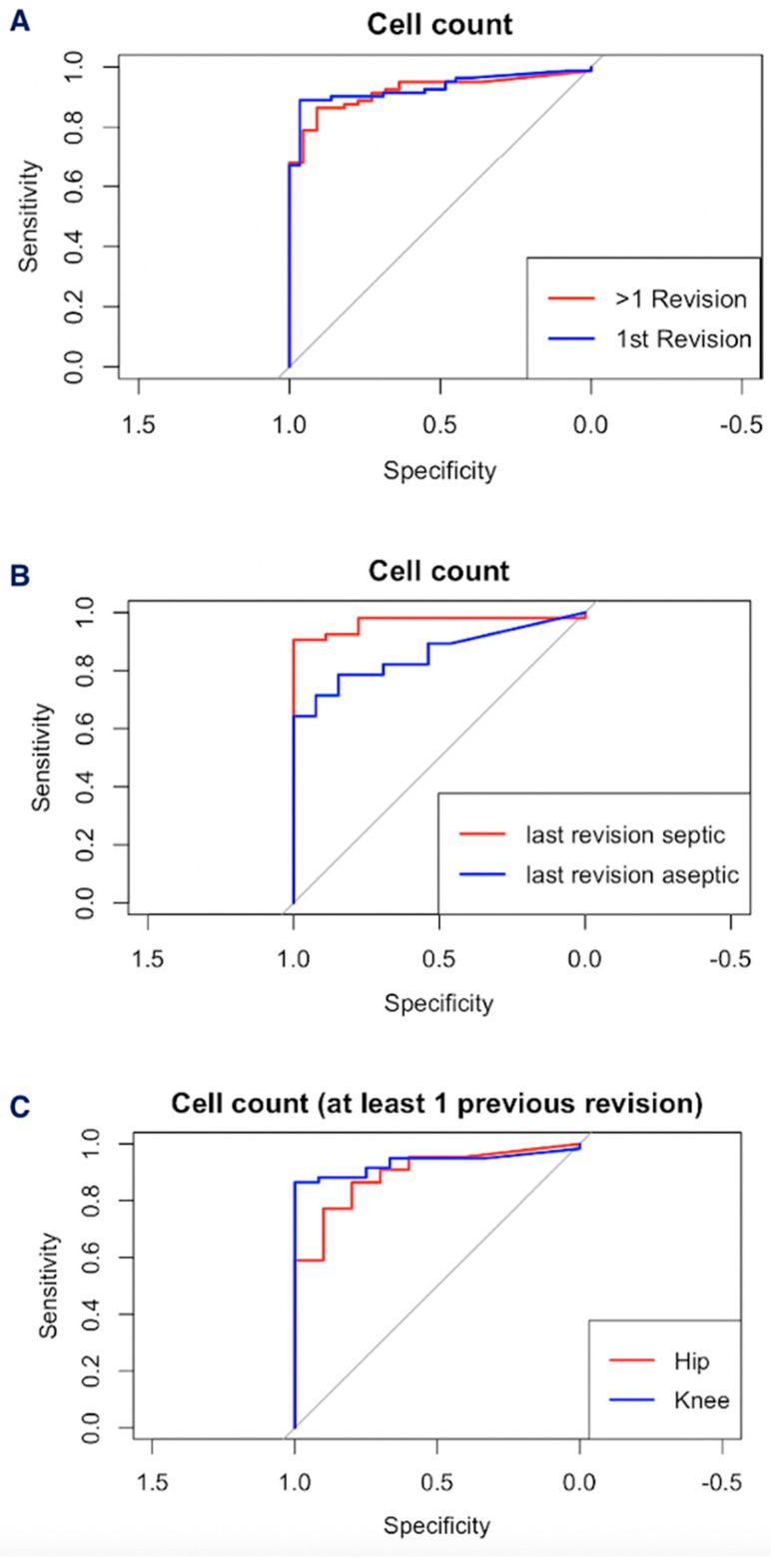
Comparison of cell count receiver operating characteristics (ROCs) depending on number of previous revisions (**A**), preceding revision type (**B**) and location (**C**).

When testing for differences in optimal cell count cutoffs between groups, we found optimal values at 3544.50 G/L (sensitivity 0.89 [0.82; 0.95], specificity 0.97 [0.90; 0.99], positive likelihood ratio (LR+) = 25.82, negative likelihood ratio (LR−) = 0.11) for patients undergoing their first revision and 2439.50 G/L (sensitivity 0.86 [0.79; 0.93], specificity 0.91 [0.77; 0.99], LR+ = 9.51, LR− = 0.15) for patients with at least one previous revision, with AUCs of 0.93 [0.88; 0.98] and 0.92 [0.87; 0.97], respectively. No significant area under the receiver operating characteristic curve (AUC) difference was found between the two groups (*p* = 0.88, Venkatraman’s test, cf. Figure 1A).

**Figure 2:**
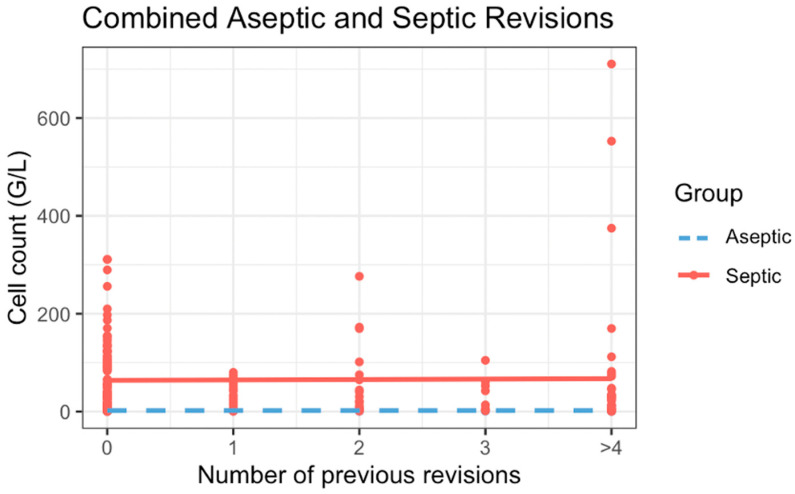
Comparison of cell count results depending on number of previous surgeries.

Neither in aseptic revisions nor in septic revisions was the cell count significantly associated with the overall number of previous surgeries (aseptic regression coefficient = 17.6, *p* = 0.95, F-statistic, and septic regression coefficient = 829.1, *p* = 0.86, F-statistic cf. Figure 2).

### 2.3. Diagnostic Cutoffs Before Re-Revision and Their Dependence on Location

Of the 103 re-revision cases, 32 hips (31.1%) and 71 knees (68.9%) were analyzed for differences in cell counts (cf. Table 3).

In terms of optimal cell count cutoffs for patients who underwent at least one revision so far, we found values for hips at 2439.5 G/L (sensitivity = 0.86 [0.72; 0.86], specificity = 0.80 [0.5; 0.99], LR+ = 4.32, LR− = 0.17) and for knees at 2626.5 G/L, with a sensitivity of 0.86 [0.75; 0.93] and specificity of 1 (LR+ was infinite due to specificity = 1, LR− = 0.14, CI = [1.00; 1.00]). AUC comparisons showed no significant difference between the two groups (hip AUC = 0.90 [0.78; 0.99], knee AUC = 0.93 [0.88; 0.99], *p* = 0.14, cf. Figure 1C).

### 2.4. Previous Septic vs. Aseptic Revision

In the event that the preceding revision surgery was septic (n = 62, 60.2%), the optimal cell count cutoff was identified at 2626.5 G/L (sensitivity = 0.91 [0.83; 0.98], specificity = 1.00, LR+ = inf., LR− = 0.09, CI = [1.00; 1.00]), with an AUC of 0.97 [0.92; 0.99]. In cases where the most recent revision was for an aseptic reason (n = 41, 39.8%), the optimal cutoff was found to be 2439.5 G/L (sensitivity = 0.79 [0.61; 0.93], specificity = 0.85 [0.62; 0.99], LR+ = 5.11, LR− = 0.25), with an AUC of 0.86 [0.75; 0.97]. The receiver operating characteristics (ROCs) for the two cohorts differed significantly (*p* = 0.03, Venkatraman’s test, cf. Figure 1B).

### 2.5. Covariate Dependent ROC Analysis

**Figure 3:**
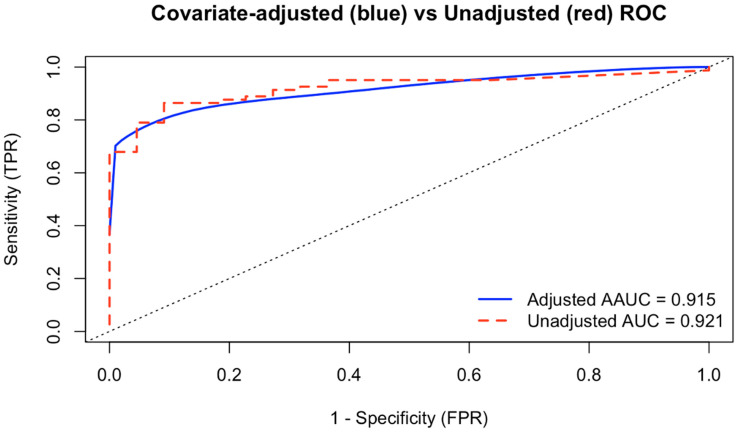
Adjusted receiver operating characteristics (ROCs) based on the joint, number of previous surgeries and type of previous revision surgery.

When applying covariate dependent ROC analysis in the re-revision cohort with the joint, type of last surgery (septic or aseptic) and the number of previous revisions (1, 2, 3 or >3) as covariates, we found an adjusted AUC (AAUC) of 0.915 (CI = [0.85; 0.96]), compared to an AUC of 0.921 for the unadjusted ROC (cf. Figure 3).

## 3. Discussion

The diagnosis of periprosthetic joint infections after revision arthroplasty remains challenging despite recent advances in diagnostics, necessitating a combination of markers to achieve accurate results. This study aimed to examine the influence of the number and type of preceding surgeries and the joint on the diagnostic thresholds for leukocyte cell counts after revision THA and TKA when applying the EBJIS criteria [13].

To the best of our knowledge, this is the first study to specifically evaluate synovial cell count in patients who underwent THA or TKA re-revision. In cases with at least one prior revision, we identified optimal cell count values at 2439.50 G/L, as opposed to 3544.50 G/L for patients who were assessed after primary implantation. Although the values slightly differ from the current diagnostic standards of 3000 G/L recommended by the EBJIS [13], synovial cell count demonstrated an excellent AUC, sensitivity and specificity, highlighting its overall reliability not only after primary THA and TKA, but also in terms of PJI assessment before re-revision.

Our findings in patients with prior revisions are further consistent with recent studies on optimal cell count cutoffs in THA and TKA, such as the 2582 G/L threshold identified by Zahar et al. [19] or the 2479 G/L reported by Gramlich et al. [15], and affirm its continued relevance and diagnostic value in patients with at least one prior revision surgery.

In addition, our study is the first to highlight that the type of immediately preceding revision surgery notably affects the diagnostic accuracy of synovial cell count. For cases with either preceding septic or aseptic revision surgery, optimal cell count cutoffs were below 3000 G/L, with 2626.5 G/L and 2439.5 G/L, respectively. However, a significantly better overall diagnostic accuracy was found for patients with previous septic revision, with a near-perfect AUC of 0.97 compared to an AUC of 0.86 for patients with directly preceding aseptic revision. Furthermore, in case of previous aseptic revision, the main reason for the four false-positive results in this cohort was a wear-induced elevation of the synovial cell count. Our findings therefore suggest that, in addition to considering the joint and mode of failure, the type of previous revision surgery should be taken into account when evaluating for PJI prior to re-revision, with a strong focus on possible false positives through wear particles and the resulting inflammatory response.

A range of studies have previously identified significantly varying cutoff values for cell counts in THA versus TKA. Regarding TKA, the recommended cutoff values range from 1100 [14] to 3600 G/L [20]. When looking at the corresponding numbers for THA, the numbers vary from 1425 G/L [21] to 4250 G/L [15], with an overall tendency towards higher values in THA compared to TKA. In patients with at least one prior revision, we identified similar trends in thresholds, with optimal values at 2439.5 G/L for rTHA and 2626.5 G/L for rTKA. Overall, the diagnostic accuracy was minimally higher for rTKAs compared to rTHA, with excellent results for both joints. Therefore, similar to what other studies have demonstrated for THA and TKA [19], our findings underline that overall diagnostic accuracy remains high for both joints, but there might be a necessity to adapt diagnostic thresholds to slightly lower cutoffs than 3000 G/L, as defined by the EBJIS criteria. Furthermore, Abdelaziz et al. previously found that 17% of patients with aseptic THA had automated leukocyte counts exceeding the 3000 G/L threshold, with the highest values associated with polyethylene wear, followed by aseptic loosening and recurrent dislocation, leading to a reduced diagnostic accuracy [22], which has also been reported in the presence of metallosis [23,24]. For TKAs, false-positive results are less of a concern, with the recent literature indicating a rate of 10.1% [25]. These findings again highlight the need for tailored diagnostic criteria and further studies assessing the impact of wear and metallosis in order to address the challenges of false positives and negatives before rTHA and rTKA.

In our analysis of cell counts, no significant differences were observed related to the number of previous revision surgeries for both septic and aseptic cases. However, we were the first to demonstrate that the type of preceding surgery had an influence on the diagnostic accuracy, with an AUC of 0.97 for patients who had a septic revision as their last surgery and an AUC or 0.86 for patients with a preceding aseptic revision. Though the differences were statistically significant, the covariate-adjusted ROC, being the average of conditional ROC curves, showed almost identical ROCs compared to the unadjusted curve for the overall cohort, supporting the overall adequacy and applicability of unadjusted synovial cell count analysis in clinical practice. Furthermore, the crossing of both the adjusted and unadjusted curve indicates that adjustments for covariates, especially the number of previous revisions, are not strictly necessary in routine clinical practice.

This study has several limitations, specifically those inherent to any retrospective single-center study. First, we were not able to additionally collect polymorphonuclear leukocyte percentages (PMN%) for a multitude of patients, and the marker was therefore not included in our analysis. Second, the findings of this study are based on automated cell count data from a single laboratory, which raises the possibility that these results may not be fully generalizable across other research centers, as differences between automated and manual cell count analysis have been reported [25]. Third, the presence of various indications for aseptic revision, such as implant loosening with proinflammatory stimuli and revisions for instability [26], resulted in a heterogeneous group of patients. This variability may have influenced our results, and larger studies are needed to further evaluate these effects. Fourth, due to the limited sample size, some diagnostic accuracy estimates reached boundary values (e.g., specificity of 1.00), reflecting the absence of false-positive cases in this cohort rather than perfect diagnostic certainty, and are therefore of limited generalizability. Fifth, the lack of routine MRSA colonization screening may have resulted in inappropriate perioperative antibiotic prophylaxis and delayed or suboptimal empirical antimicrobial treatment.

## 4. Materials and Methods

### 4.1. Study Design

This study received approval from the local ethics committee (Nr.1259/2021). It was a retrospective single-center study of patients who underwent revision THA or TKA from June 2012 to May 2024 as documented by our arthroplasty registry. Our database contains automated leukocyte cell counts from synovial fluid and includes results from intraoperative microbiological samples, sonication and histological analysis, all following the EBJIS guidelines [13]. Further demographics such as age at time of surgery, sex and comorbidities according to the Charlson Comorbidity Index (CCI) [27] were collected. In order to avoid incorporation bias, we opted to exclude leukocyte cell count as a criterion for distinguishing between septic and aseptic cases based on the EBJIS criteria. Two investigators (J. Straub and P.M. Schwarz) independently evaluated all cases for fulfillment of the inclusion criteria, with discrepancies resolved by consensus to minimize selection bias. Furthermore, acute postoperative infections less than 90 days from last surgery were excluded. Histologic samples were graded using the synovial-like interface membrane (SLIM) classification [28]. Depending on the postoperative course after reimplantation, the cases were classified using the MSIS outcome reporting tool [29].

In terms of antibiotic prophylaxis in revision surgery at our institution, patients receive two weeks of intravenous antibiotics after explantation, followed by four weeks of oral antibiotics as prescribed by our infectious disease specialists, depending on the respective antibiogram. Spacers with vancomycin ant gentamicin cement are used by default. After the second stages, patients received another two weeks of intravenous antibiotics, with additional fosfomycin or rifampin as biofilm active agents. Hereafter, four to ten weeks of oral antibiotics were prescribed. For one-stage exchanges, surgery was followed by two weeks of intravenous antibiotics, followed by four to ten weeks of oral antibiotics after consultation with our infectious disease specialists. In primary implantations, at least one single-shot antibiotic treatment with weight-adjusted cefazolin is administered, which is prolonged depending on the surgeon’s instructions.

Antibiotics were routinely administered only after joint aspiration was performed. Patients were not routinely screened for MRSA colonization pre- or post-operatively.

### 4.2. Statistics

Demographic variables were analyzed descriptively through median and quartile values for metric variables and absolute numbers along with percentages for categorical variables. Metric variables were compared using *t*-tests or Wilcoxon signed-rank tests depending on the respective distribution. For categorical variables, a Chi-square test was employed.

For each subgroup (first revision vs. re-revision, last revision septic vs. last revision aseptic, hip vs. knee), sensitivity, specificity and area under the receiver operating characteristic curve were calculated, as well as negative and positive likelihood ratios. Optimal cell count thresholds were determined for each subgroup through ROC analysis based on the Euclidean distance between the ROC curve and the (0, 1) point. AUCs between groups were compared with the Venkatraman test for unpaired samples.

Additionally, we performed a covariate-dependent ROC analysis using the nonparametric Bayesian approach for re-revision cases, with the joint, the type of last surgery (septic or aseptic) and the number of previous revisions (1, 2, 3 or >3) as covariates.

Furthermore, correlations between time from last surgery and cell count, as well as the number of previous surgeries and cell count, were assessed through linear regression. All tests were performed in their two-sided versions at a significance level of 0.05 using R (Version 4.2.2) [30].

### 4.3. Demographics

A total of 214 patients were included in our final analysis, comprising 139 rTKAs (64.9%) and 75 rTHAs (35.1%). Among these patients, 96 were male (44.9%) and 118 were female (55.1%). In total, 163 cases (76.2%) were considered septic revisions and 51 (23.8%) aseptic according to the EBJIS criteria without leukocyte count (cf. Table 1).

## 5. Conclusions

In conclusion, the synovial leukocyte count demonstrates an excellent diagnostic accuracy not only in primary revisions, but also for patients undergoing re-revision. Though the type of preceding surgery was shown to have an influence on diagnostic accuracy, and minor threshold variations from the current EBJIS criteria were found, the overall diagnostic performance remains high. This was underscored by a covariate-adjusted ROC analysis, with almost identical results compared to the unadjusted curve, indicating a high robustness and overall diagnostic value.

## Figures and Tables

**Table 1:** Overview of demographics described via absolute numbers (%) and median (IQR).

	First Revision (n = 111)	Re-Revision (n = 103)	*p*-Value
THAs	43 (38.7%)	32 (31.1%)	0.30
TKAs	68 (61.3%)	71 (68.9%)	
Male	48 (43.2%)	48 (46.6%)	0.72
Age	73.0 [64.0; 80.0]	76.0 [67.0; 79.5]	0.23
CCI	4 [3.0; 5.0]	4.0 [3.0; 5.0]	0.58
Diabetes	22 (19.8%)	21 (20.4%)	0.99
EBJIS criteria	82 (73.8%)	81 (78.6%)	0.51
Time since last revision (months)	50.3 [12.1; 162.8]	11.7 [3.3; 41.1]	**0.0002**

Abbreviations: Total hip arthroplasties (THAs), total knee arthroplasties (TKAs), Charlson Comorbidity Index (CCI). Bold values indicate significant results.

**Table 2:** Overview of positive culture results for revision and re-revision cases.

Positive Culture Results	First Revision (n = 63)	Re-Revision (n = 65)
*Staphylococcus epidermidis*/ *Coagulase-negative* *staphylococci*	34 (26.6%)	18 (27.7%)
*Staphylococcus aureus*	33 (25.8%)	19 (29.2%)
*Multiple pathogens*	14 (10.9%)	9 (13.8%)
*β-hemolytic streptococci*	7 (5.5%)	2 (3.1%)
*Cutibacterium acnes*	7 (5.5%)	3 (4.6%)
*Candida* spp.	6 (4.7%)	4 (6.2%)
*Proteus mirabilis*	4 (3.1%)	2 (3.1%)
*Escherichia coli*	4 (3.1%)	1 (1.5%)
*Corynebakterium* spp.	3 (2.3%)	2 (3.1%)
*Enterococcus faecalis*	3 (2.3%)	2 (3.1%)
*Streptococcus anginosus*	2 (1.6%)	0 (0.0%)
*Steptococcus mitis*	2 (1.6%)	1 (1.5%)
*Moraxella osloensis*	1 (0.8%)	0 (0.0%)
*Listeria monocytogenes*	1 (0.8%)	1 (1.5%)
*Enterococcus faecium*	1 (0.8%)	0 (0.0%)
*Enterobacter cloacae*	1 (0.8%)	1 (1.5%)
*Parvimonas micra*	1 (0.8%)	0 (0.0%)
*Streptococcus agalactiae*	1 (0.8%)	0 (0.0%)
*Streptococcus dysgalactiae*	1 (0.8%)	0 (0.0%)
*Streptococcus gordonii*	1 (0.8%)	0 (0.0%)
*Streptococcus pneumoniae*	1 (0.8%)	0 (0.0%)

**Table 3:** Overview of cell counts in re-revision total hip arthroplasty and re-revision total knee arthroplasties compared between septic and aseptic cases, described through median and interquartile range (IQR).

	Re-Revision Total Hip Arthroplasty (n = 32)	Re-Revision Total Knee Arthroplasty (n = 71)	*p*-Value
Aseptic revision synovial cell count (G/L)	1180 [1000; 1520]	1108 [1000; 2058]	0.92
Septic revision synovial cell count (G/L)	32,220 [5653; 67,660]	20,165 [4438; 43,219]	0.21

## Data Availability

The participants of this study did not give written consent for their data to be shared publicly, so due to the sensitive nature of the research supporting data is not available.

## Abbreviations

The following abbreviations are used in this manuscript:

THA	Total Hip Arthroplasty
TKA	Total Knee Arthroplasty
rTHA	Revised Total Hip Arthroplasty
rTKA	Revised Total Knee Arthroplasty
EBJIS	European Bone and Joint Infection Society
ROC	Receiver Operating Curve
PJI	Periprosthetic Joint Infection
AUC	Area Under the Curve
AAUC	Adjusted Area Under the Curve
SLIM	Synovial-Like Interface Membrane
MSIS	Musculoskeletal Infection Society
LR+	Positive Likelihood Ratio
LR−	Negative Likelihood Ratio
CCI	Charlson Comorbidity Index
